# CHAF1A promotes the proliferation and growth of epithelial ovarian cancer cells by affecting the phosphorylation of JAK2/STAT3 signaling pathway

**DOI:** 10.1016/j.bbrep.2023.101522

**Published:** 2023-07-31

**Authors:** Dandan Xia, Xun Xu, Jing Wei, Wenli Wang, Jiali Xiong, Qingqing Tan, Pingping Xue, Huiyan Wang

**Affiliations:** a Department of Obstetrics and Gynecology, Changzhou Maternal and Child Health Care Hospital, Changzhou Medical Center, Nanjing Medical University, Changzhou, 213000, China; b Department of Orthopedics, Changzhou Maternal and Child Health Care Hospital, Changzhou Medical Center, Nanjing Medical University, Changzhou, 213000, China; c Department of Reproductive Medicine Center, Changzhou Maternal and Child Health Care Hospital, Changzhou Medical Center, Nanjing Medical University, Changzhou, 213000, China

**Keywords:** 【Key words】CHAF1A, Epithelial ovarian cancer, JAK2, STAT3, Proliferation, Apoptosis

## Abstract

The molecular mechanism of chromatin assembly factor 1 unit A (CHAF1A) promoting the proliferation and growth of epithelial ovarian cancer (EOC) cells hasn’t been reported at present. In this study, recombinant CHAF1A siRNA/overexpression plasmid (si-RNA1/pcDNA3.1-CHAF1A) was designed and constructed, and stable cell lines with knockdown or overexpression of CHAF1A were constructed. The changes of JAK2/STAT3 pathway were detected by Western blot. JAK2/STAT3 pathway was inhibited by Peficitinib, and then cell proliferation and growth ability were detected. Bioinformatics analysis suggested that CHAF1A was up-regulated in epithelial ovarian cancer. JAK2/STAT3 pathway phosphorylation was inhibited in si-RNA1 group, while it was increased in pcDNA3.1-CHAF1A group. After inhibiting JAK2/STAT3 pathway, the promoting effect of CHAF1A on epithelial ovarian cancer cell proliferation disappeared, meanwhile the inhibitory effect of CHAF1A on apoptosis enhanced. In conclusion, CHAF1A promotes the proliferation and growth of epithelial ovarian cancer cells by affecting the phosphorylation of JAK2/STAT3 signaling pathway.

## Highlights

• CHAF1A can activate the phosphorylation of JAK2/STAT3 signaling pathway.
• Inhibition of JAK2/STAT3 signaling pathway could reduce the effect of CHAF1A overexpression on the proliferation and growth of epithelial ovarian cancer cells.

## 1 Introduction

As one of the three major malignant tumors of the female reproductive system, ovarian cancer ranks first among gynecological malignancies with a mortality rate of up to 70% [1]. Epithelial Ovarian Cancer (EOC) accounts for 85%–90% of ovarian malignant tumors [2], and the current treatment is mainly surgery and adjuvant chemotherapy. The cure rate of early-stage ovarian cancer is up to 90%. However, the lack of specific signs and symptoms in the early stages of the disease is due to being anatomically hidden deep in the pelvic cavity, so when diagnosed more than half of patients are already at an advanced stage, and the five-year survival rate of patients with stage III-IV is only 30% [3]. Therefore, the development of novel drugs to block tumor proliferation, invasion, metastasis and recurrence is of great importance to provide important targets for improving survival rate, improving prognosis, and reducing recurrence rate. Chromatin Assembly Factor −1 subunit A (CHAF1A) is a key gene responsible for connecting cell cycle progresses and activating DNA damage checkpoints [4,5]. Functional studies have found that CHAF1A is involved in chromatin loading, DNA replication, gene expression regulation and DNA missense repair. In addition to enriching gene sets for DNA damage repair, DNA metabolism, and chromatin dynamic regulation, CHAF1A also regulates gene sets related to cell cycle, DNA replication, and cytoskeleton regulation [6]. Many studies suggest that it may be highly expressed in a variety of tumors, which can affect the proliferation, metastasis and invasion of tumor cells. Previous studies of our group have shown that CHAF1A acts as a growth promoting agent in epithelial ovarian cancer, and its high expression is associated with clinical stage and lymph node metastasis, which can promote cancer cells proliferation and inhibit cancer cells apoptosis [7]. In this study, si-RNA and overexpressed plasmid of CHAF1A in epithelial ovarian cancer cells was used to explore the molecular mechanism of CHAF1A, and mainly discusseed whether JAK2/STAT3 signaling pathway is involved,in order to provide a theoretical basis for the prognosis and treatment of ovarian cancer patients.

## 2 Materials and methods

### 2.1 Materials

Human epithelial ovarian cancer cell lines HO8910 and SKOV3 were purchased from the cell bank of Shanghai Chinese Academy of Sciences. Cell culture conditions: HO-8910:RPMI-1640 + 10% FBS+1% P/S; SKOV3: McCoy's 5A + 10% FBS+1% P/S. RPMI 1640 medium, fetal bovine serum, penicillin/streptomycin double antibody, puromycin, Triazole, SYBR fluorescence quantitative PCR Kit,Script Ⅳ First⁃Strand kit, RIPA lysate (Thermos, USA); BCA protein detection kit, crystal violet solution (Hangzhou Beyotime Biological Company); rabbit anti-human Bcl-2 monoclonal antibody, rabbit anti-human Caspase-3 monoclonal antibody, rabbit anti-humanβ⁃actin monoclonal antibody (proteintech, USA), rabbit anti-human JAK2 monoclonal antibody, rabbit anti-human p⁃JAK2 monoclonal antibody, rabbit anti-human STAT3 Monoclonal antibody, Rabbit anti-human p⁃STAT3 monoclonal antibody, Sheep anti-Rabbit IgG antibody labeled by HRP (Abcam, UK); Lenti⁃Pac Lentivirus Packaging Kit (Genocopoeia, USA); CCK⁃8 kit (Tongram Corporation, Japan); JAK2/STAT3 inhibitor peficitinib (Gene Operation Company, USA).

### 2.2 Methods

#### 2.2.1 Western blot analysis

The total protein was extracted by RIPA lysis solution and the protein concentration was determined by BCA method. The total protein was mixed with sample loading buffer in equal proportion, heated at 95 °C for 10 min, and stored at −20 °C. The protein samples were subjected to polyacrylamide gel electrophoresis and transferred to PVDF membrane. 5% skim milk powder was enclosed at room temperature for 2 h and incubated overnight with primary antibody (1:5000) at 4 °C. PBST was rinsed for 3 times, and HRP labeled sheep anti-rabbit IgG antibody (1:5000) was incubated for 1 h. The PBST was rinsed 3 times and the SuperSignal exposure solution for ChemiDoc XRS + system exposure covered the surface of the film. The gene expression levels were calculated using the 2-ΔΔCq method. The experiment was repeated three times.

#### 2.2.2 Quantitative reverse transcriptase-polymerase chain reaction (qRT-PCR)

Total RNA was extracted from logarithmic growth cells by Triazole method, RNA concentration and purity were determined by One Drop OD 1000, and cDNA was obtained by reverse transcription using Super⁃ Script Ⅳ First⁃Strand kit. SYBR kit was used for sample amplification in StepOnePlus real-time PCR system. Amplification conditions were 95 °C for 10 min. 95 °C for 15 s, 56 °C for 1 min, 30 cycles; 95 °C 15 s, 60 °C 1 min, 95 °C 15 s. The primers were synthesized by Jiangsu Zhiyuan Decoding Biotechnology Co. LTD. Using GAPDH as reference gene. All experiments were repeated three times.

#### 2.2.3 Construction and identification of CHAF1A knockdown/overexpression plasmid

The siRNAs used in the study are listed in Table 1. The pcDNA3.1-CHAF1A or pcDNA3.1 vector plasmids were purchased from TsingkeBiotechnologyCo.,Ltd(Beijing, China). The full length of CHAF1A is 2883bp. Cells were cultured in six-well plates and then transfected with siRNA or plasmid using Lipofectamine 2000 (Invitrogen, Carlsbad,CA, USA), following the manufacturer protocol.

**Table 1:** The sequences for CHAF1A siRNA.

Gene	Gene ID	sequence(5'-3')	Antisense(5'-3')
CHAF1A	NM-005483.3	GGUUAAGAGAAGAAGAGAA	UUCUCUUCUUCUCUUAACC
		GGAAGAAGAGAAACGGUUA	UAACCGUUUCUCUUCUUCC
		GCUCUACAGAGAAGAACAA	UUGUUCUUCUCUGUAGAGC

#### 2.2.4 Cell transfection and screening of stable strains

Each plasmid was transfected into 293T cells using Lenti⁃Pac lentivirus. After 48 h of culture, the supernatant was collected, centrifuged and filtered, mixed with concentrated reagent in proportion, and incubated at 4 °C overnight. The next day, the mixture was centrifuged and lentiviral particles were collected. HO8910 and SKOV3 cells were inoculated into 24-well plates, and lentiviral particles were added 24 h later to infect target cells. Cells transfected with no-load constitution plasmid were labeled pc-DNA3.1, cells transfected with different knockdown CHAF1A plasmids were labeled si-RNA1, and cells transfected with overexpressed CHAF1A plasmids were labeled pcDNA3.1-CHAF1A. 72 h later, puromycin was added (0.75 μg/mL) and cultured for 1 week, and puromycin was reduced (0.35 μg/mL) for 2 weeks.

#### 2.2.5 CCK-8 proliferation experiment

Cells in each group were planted in 96-well plates with 1 × 104 cells per well, each group was provided with 3 multiple Wells, and cell-free medium was used as blank group. The original medium was removed at 24, 48, 72 and 96 h, and 10 μLCCK-8 reagent and 90 μL medium were added to each well. The medium was incubated at 37 °C for 3 h under light protection. The light absorption values of each well at 450 nm wavelength were detected by enzyme labeling. The experiment was repeated three times.

#### 2.2.6 Colony formation assays

500 treated log-phase cells were suspended and then plated on to 6-well plates to evaluate the colony-formation ability. Cells were cultured in an incubator for 14 days. After fixation, the colonies were stained by Giemsa solution. Colonies were then counted and photographed.

#### 2.2.7 Flow cytometry

After transfection, cells in the logarithmic growth phase were washed three times with PBS. Suspended cells at a concentration of 1 * 10^6^/ml were fixed with 70% ethanol at 4 °C overnight. Following propidium iodide (PI) staining, DNA content was measured using flow cytometry (BD Biosciences, San Jose, CA, USA). The cellular apoptosis assay was performed by using the PI/Annexin V-FITC Apoptosis Kit (Sigma, USA) according to the manufacturer's instructions. A FACS can flow cytometer and FlowJo software (Tree Star Inc., Ashland, OR) were used to analyze the staining data.

### 2.3 Statistical methods

We used SPSS 22.0 statistical software to analyze the data, and the measurement data were expressed as mean ± standard deviation (x ± s). t -test was used for mean comparison between two groups; one-way ANOVA was used for comparison between groups; SNK ⁃ q test was used for pairwise comparison; P ≤ 0.05 was considered as statistically significant difference.

## 3 Results

### 3.1 Results of bioinformatics analysis

**Fig. 1:**
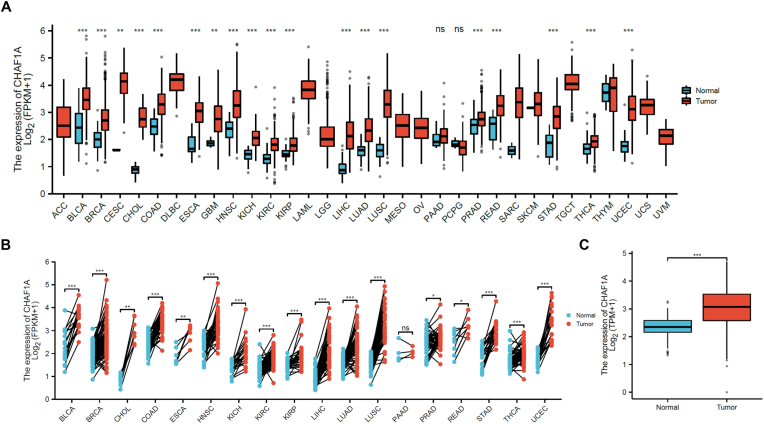
Analysis of bioinformatics results. (A)The expression of CHAF1A was significantly up-regulated in most tumors in pan-carcinoma unpaired samples. (B) The expression of CHAF1A was significantly up-regulated in most tumors in pan-carcinoma paired samples. (C)In unpaired samples, CHAF1A expression was significantly up-regulated in ovarian cancer.

The expression of CHAF1A was significantly up-regulated in most tumors in pan-carcinoma unpaired samples by bioinformatics analysis (Fig. 1A), and significantly up-regulated in most tumors in pan-carcinoma paired samples (Fig. 1B). In unpaired samples, CHAF1A expression was significantly up-regulated in ovarian cancer (Fig. 1C). These results confirmed the oncogene role of CHAF1A in malignant tumors including EOC,which are consistent with previous literature reports and our previous experimental results.

### 3.2 CHAF1A enhanced the activation of JAK2/STAT3 signaling pathway

**Fig. 2:**
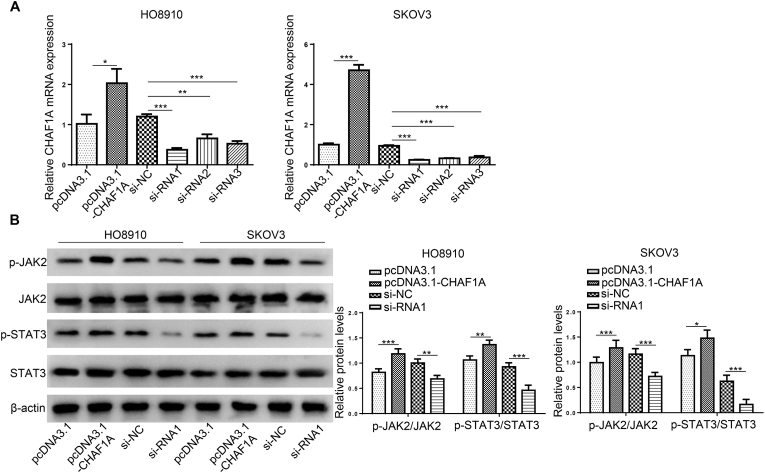
CHAF1A enhances activation of the JAK2/STAT3 signaling pathway. (A) The addition of overexpressed plasmids pcDNA3.1-CHAF1A and knockdown si-RNA-CHAF1A to HO8910 and SKOV3, respectively, and qRT⁃PCR to demonstrate the validity of stable transfection (***P < 0.001,**P < 0.01, *P < 0.05). (B) Western blot showed the phosphorylation level of JAK2/STAT3 after the increase and knockdown of CHAF1A in HO8910 and SKOV3 cells (***P < 0.001,**P < 0.01, *P < 0.05).

First, we constructed CHAF1A overexpression/knockdown stable cell lines. The overexpressed plasmids pcDNA3.1-CHAF1A and knock-down si-RNA-CHAF1A were added to HO8910 and SKOV3, respectively, to demonstrate the effectiveness of stable transfection using qRT⁃PCR (***P < 0.001,**P < 0.01, *P < 0.05,Fig. 2A). In this study, si⁃RNA 1 with higher efficiency was selected from the knockdown strains for further study. The results showed that after the overexpression of CHAF1A in HO8910 and SKOV3 cells, the phosphorylation level of JAK2/STAT3 was increased by Western blot detection, and the phosphorylation level of JAK2/STAT3 in the cells was decreased after the knockdown of CHAF1A (***P < 0.001,**P < 0.01, *P < 0.05, Fig. 2B).

### 3.3 Inhibiting the activation of JAK2/STAT3 can reduce the proliferative effect of CHAF1A

**Fig. 3:**
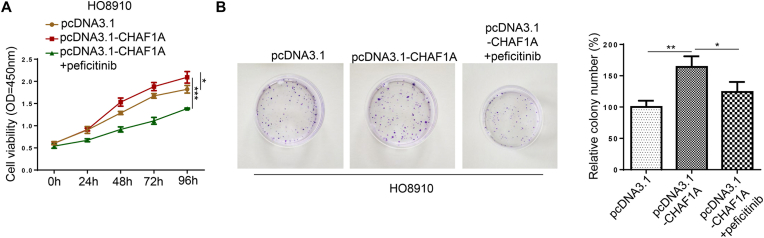
Inhibition of JAK2/STAT3 activation can reduce the proliferative effect of CHAF1A. (A) CCK⁃8 assay was used to test the cell proliferation rate of after HO8910 cells cultured with peficitinib (4.8 nmol/L), a JAK2/STAT3 pathway inhibitor (***P < 0.001, *P < 0.05). (B) As assessed by colony formation, the pcDNA3.1-CHAF1A + peficitinib group was lower than that of pcDNA3.1-CHAF1A group (**P < 0.01, *P < 0.05).

The JAK2/STAT3 pathway inhibitor,peficitinib (4.8 nmol/L), was added into HO8910 cells cultured with pcDNA3.1-CHAF1A. The cell proliferation rate of pcDNA3.1-CHAF1A + peficitinib group was lower than that of pcDNA3.1 and pcDNA3.1-CHAF1A groups according to CCK-8 assay results (***P < 0.001, *P < 0.05, Fig. 3A). The clone formation rate of pcDNA3.1-CHAF1A + peficitinib group was lower than that of pcDNA3.1-CHAF1A group (**P < 0.01, *P < 0.05, Fig. 3B).

### 3.4 Inhibiting the activation of JAK2/STAT3 can reduce the anti-apoptotic effect of CHAF1A

**Fig. 4:**
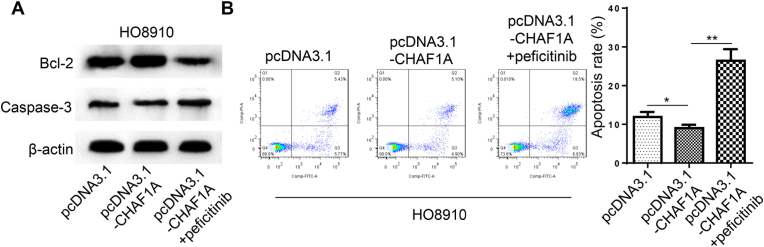
Inhibition of JAK2/STAT3 activation can reduce the anti-apoptotic effect of CHAF1A. (A) Western blot showed that the expression of anti-apoptotic protein Bcl-2 in pcDNA3.1-CHAF1A + peficitinib group was down-regulated, while the expression of pro-apoptotic protein Caspase-3 was up-regulated. (B)Flow cytometry showed that the apoptosis rate of pcDNA3.1-CHAF1A + peficitinib group was higher than that of pcDNA3.1 and pcDNA3.1-CHAF1A group (**P < 0.01, *P < 0.05).

Peficitinib (4.8 nmol/L), was added into HO8910 cells cultured with pcDNA3.1-CHAF1A again. Western blot detected the expression of apoptosis related proteins at the protein level. pcDNA3.1-CHAF1A + peficitinib group down-regulated the expression of anti-apoptotic protein Bcl-2, and up-regulated the expression of pro-apoptotic protein Caspase-3 (Fig. 4A). The apoptosis rate of pcDNA3.1-CHAF1A + peficitinib group was higher than that of pcDNA3.1 and pcDNA3.1-CHAF1A groups showed by Flow cytometry (**P < 0.01, *P < 0.05, Fig. 4B).

## 4 Discussion

Chromatin Assembly Factor −1 (CAF-1) is the only known histone chaperone that recruits histones H3 and H4 into newly synthesized DNA, which is a heterotrimer composed of P150, P60, and P48. It mainly mediates the decomposition and recombination of nucleosomes, promotes the rapid assembly of nucleosomes, participates in DNA assignment, and play a crucial role in DNA damage repair and heterochromatin formation. As a core component of CAF-1, subunit P150/Chromatin Assembly Actor 1 subunit A (CHAF1A), is a key gene for activating DNA damage checkpoints and regulating cell-cycle progression. It can regulate the expression of a series of downstream genes and repair oxidative base damage, as a very important factor in DNA replication. Many studies suggest it may be highly expressed in in many malignant tumors, participate in tumor proliferation, invasion, differentiation and other tumor phenotypes, and is closely associated with malignant progression and adverse survival outcomes of a variety of human tumors. CHAF1A is overexpressed in gastric cancer cell lines and tissue samples, and its high expression predicts poor prognosis [8]. In addition, studies have demonstrated that CHAF1A may affect the efficacy of adjuvant chemotherapy in gastric cancer by regulating the expression of thymidylate synthase [9]. The expression of CHAF1A was positively correlated with the tumor size and clinical stage of glioblastoma, and negatively correlated with the overall survival rate, which could be used as an independent biological marker to estimate the prognosis [10]. Meanwhile, CHAF1A has also been found to play an important role in colon cancer, breast cancer and other tumors, which is related to cell proliferation, metastasis, apoptosis and poor prognosis of patients [11]. However, there was no literature report on the relationship between CHAF1A and ovarian cancer before our study.

The cancer genome atlas (TCGA) is a large-scale cancer research project initiated in the United States in 2006. Currently, TCGA has received more and more attention, and has been widely used in tumor-related mutation analysis, copy number analysis, mRNA and miRNA expression, DNA methylation analysis and signaling pathway analysis [12,13]. We found that compared with normal ovarian epithelial tissues, CHAF1A was highly expressed in epithelial ovarian cancer tissues and correlated with patient stage upgrading and lymph node metastasis by analyzing TCGA database and combining with our published articles. Similarly, we also found high expression of CHAF1A in epithelial ovarian cancer cell lines. Further cytofunctional experiments showed that CHAF1A could promote the proliferation and inhibit cell apoptosis of epithelial ovarian cancer cells [7]. We further investigated the downstream pathways affected by CHAF1A in epithelial ovarian cancer.

It was suggested that CHAF1A could affect cell proliferation and apoptosis through AKT/FOXO3a/Bim pathway in glioblastoma [10]. In the study of bladder cancer, human gene expression profiling microarray sequencing combined with Ingenuity Pathway Analysis (IPA) were used to explore the changes of downstream gene expression and related signaling pathways in bladder urothelial carcinoma after CHAF1A knockdown. These differentially expressed genes were enriched in signaling pathways related to cell growth and proliferation, such as MAPK pathway, PI3K/AKT pathway, ERK pathway and JAK/STAT pathway, which have been found that play very important roles in the occurrence and development of tumors [14]. In addition, in bladder cancer cell J82 with down-regulated expression of CHAF1A, some down-regulated expression of several genes related to cell growth and proliferation were found, including STAT2, STAT6, AKT2, IRS1 and SOX4.

Our study found that CHAF1A was involved in the expression of a variety of genes related to cell proliferation and apoptosis (Caspase-3, Bcl-2), which are downstream molecules of JAK2/STAT3 signaling pathway, indicating that CHAF1A may affect epithelial ovarian cancer cells through JAK2/STAT3 signaling pathway. The Janus kinase/signal transducer and activator of tragions (JAK/STAT) signaling pathway is a newly discovered signal transduction pathway stimulated by cytokines. It is involved in many important biological processes such as cell proliferation, differentiation, apoptosis and immune regulation [15,16]. STAT3, as the most important research member, promotes transcription by the following mechanisms [17]: Cytokines and growth factors interact with receptors to bring receptor-coupled JAK2 kinases close to each other and activate them through interactive tyrosine phosphorylation. Activated JAK2 kinases stimulate the tyroramate phosphorylation of the recipient body and form corresponding STAT3 docking sites. STAT3 binds to the binding domain of the docking site of the receptor STAT3 via the SH2 domain, which is phosphorylated by the JAK2 kinase. Then the phosphorylated STAT3 forms a homodimer/heterodimer and acts as a second messenger into the nucleus. Binding to the upstream promoter of the corresponding target gene initiates the expression of specific target genes, transcription and translation of specific proteins to regulate cell proliferation, differentiation and apoptosis [18]. Studies have shown that abnormal activation and overexpression of STAT3 exist in a variety of tumor tissues and cells, and up-regulate the expression of genes related to cell proliferation, apoptosis, angiogenesis, invasion and metastasis, which plays an important role in the occurrence and development of tumor [19]. Continuous activation of JAK2/STAT3 signaling pathway is often associated with the proliferation, invasion, metastasis and other behaviors of malignant tumors [20], which is also the focus of research on JAK/STAT signaling pathway.

Current studies have shown that STAT activation can be detected in all ovarian cancers, especially STAT3, which is associated with tumor occurrence, invasion and metastasis, suggesting that JAK/STAT signaling pathway can support ovarian cancer cell survival by inhibiting or inducing genes involved in cell survival and apoptosis. STAT3 is hyperactive in ovarian cancer, and increased p-STAT3 levels are mediated by increased reactive oxygen species production in hypoxic cancer cells. Inhibition of STAT3 with AG490 followed by treatment with cisplatin or paclitaxel resulted in a significant increase in apoptosis, suggesting that hypoxia-induced activation of STAT3 is associated with chemotherapy resistance, suggesting that STAT3 has a tendency to promote drug resistance in ovarian cancer [21]. Current studies have suggested that the enhancement of JAK/STAT pathway activity can promote the proliferation, growth, invasion and metastasis of ovarian cancer cells, inhibit cell apoptosis, and progress towards cisplatin resistance [22]. Therefore, it is an important target for the treatment of ovarian cancer and the prevention of cisplatin resistance in ovarian cancer therapy.

This study demonstrated that the phosphorylation of JAK2/STAT3 in si-RNA1 cells with knockdown of CHAF1A was decreased, and the phosphorylation of JAK2/STAT3 in pcDNA3.1-CHAF1A cells with overexpression of CHAF1A was increased. These results indicated that CHAF1A could promote the phosphorylation of JAK2/STAT3 signaling pathway. In addition, after inhibiting JAK2/STAT3 signaling pathway activation with peficitinib, the proliferation rate of pcDNA3.1-CHAF1A cells was decreased, the apoptosis rate was increased, the expression of pro-apoptotic protein Caspase-3 was up-regulated, and the expression of anti-apoptotic protein Bcl-2 was down-regulated. These results suggest that inhibition of JAK2/STAT3 signaling pathway could reduce the effect of CHAF1A overexpression on the proliferation and growth of epithelial ovarian cancer cells. These results indicate that CHAF1A affects the biological behavior of epithelial ovarian cancer cells and regulates the expression of downstream proteins Caspase-3 and Bcl-2 by promoting JAK2/STAT3 signaling pathway phosphorylation.

However, there were several limitations in the present study. Firstly, HO-8910 might be a problematic cell line, and the present findings should be validated using additional EOC, such as Hey and other cell lines to ensure a more in-depth analysis. Secondly, it is necessary to validate the expression of CHAF1A in a larger cohort, and analyze the relationship between CHAF1A and prognosis of ovarian cancer in future study.

CHAF1A promotes the proliferation and growth of epithelial ovarian cancer cells and inhibits the apoptosis of cancer cells by activating JAK2/STAT3 signaling pathway, which is expected to be a new target for the treatment of epithelial ovarian cancer. However, the specific way CHAF1A affects the JAK2/STAT3 pathway still needs to be further studied.

## Declaration of competing interest

The authors declare that they have no conflicts of interest.

## Data Availability

No data was used for the research described in the article.
